# Incidence of new onset arterial hypertension after metabolic bariatric surgery: an 8-year prospective follow-up with matched controls

**DOI:** 10.1097/HJH.0000000000003993

**Published:** 2025-03-10

**Authors:** Viiko Vahtera, Jukka S. Pajarinen, Mika Kivimäki, Jenni Ervasti, Jaana Pentti, Sari Stenholm, Jussi Vahtera, Paulina Salminen

**Affiliations:** a Päijät-Häme Central Hospital, Department of Surgery, Lahti; b Department of Surgery, University of Turku, Turku; c Department of Plastic and Reconstructive Surgery, University of Helsinki and Helsinki University Central Hospital, Helsinki; d Finnish Institute of Occupational Health, Finland; e UCL Brain Sciences, University College London, London, UK; f Clinicum, Faculty of Medicine, University of Helsinki; g Department of Public Health; h Centre for Population Health Research, University of Turku and Turku University Hospital, Turku, Finland; i Research Services, Turku University Hospital and University of Turku; j Division of Digestive Surgery and Urology, Turku University Hospital, Turku, Finland

**Keywords:** cardiovascular risk prevention, metabolic bariatric surgery, obesity and arterial hypertension, propensity score

## Abstract

**Background:**

Metabolic bariatric surgery (MBS) reduces the risk of new-onset hypertension; however, it is unclear whether this effect varies according to patient sex, age, or socioeconomic background. This study aimed to assess the risk of new-onset arterial hypertension after MBS, with a special focus on these patient characteristics.

**Methods:**

This follow-up study with matched controls was nested in a large employee cohort, the Finnish Public Sector study, consisting of individuals with no hypertension at baseline. For each patient who underwent laparoscopic MBS between 2008 and 2016, two propensity-score matched controls were selected from individuals hospitalized with a diagnosis of obesity or individuals with self-reported severe obesity [body mass index (BMI) ≥ 35 kg/m^2^] but no recorded history of MBS. Cases of new-onset hypertension were identified via linked electronic health records from the national health registries until December 31, 2016.

**Results:**

The study included 912 patients and 1780 matched controls. The rate of new-onset hypertension per 1000 person-years was 2.8 in the surgery group and 9.6 in the control group, with a rate ratio of 0.29 (95% confidence intervals 0.15–0.57) and a rate difference of −6.8 (95% confidence intervals −9.6 to −4.0) per 1000 person-years. No significant differences in rate reduction after MBS were observed to be associated with patient sex, age, or socioeconomic status.

**Conclusion:**

Metabolic bariatric surgery reduces the risk of new-onset arterial hypertension across all age-, sex-, and socioeconomic subgroups.

## INTRODUCTION

Arterial hypertension is a leading cause of cardiovascular mortality [1,2]. In 2023, it was estimated that 33% of the global adult population had hypertension, defined as systolic blood pressure (PB) ≥140 mmHg and/or diastolic BP ≥90 mmHg [3]. The prevalence of hypertension has increased and is expected to continue to increase, especially in low- and middle-income countries [3], contributing to increasing total deaths from cardiovascular diseases worldwide, underlining the importance of sustainable interventions for reducing hypertension [3–6]. At the same time, severe obesity is an increasing global epidemic with estimations that 20% of the global population will suffer from obesity (BMI ≥ 30 kg/m^2^) by year 2030 [7,8]. Obesity is a leading predisposing factor for several chronic conditions with major public health importance including hypertension, type 2 diabetes (T2DM), coronary heart disease, and stroke [7,9–12]. Both hypertension and obesity are major modifiable risk factors for cardiovascular disease and all-cause mortality [2,5,13,14]. The pathogenesis of severe obesity and hypertension is linked, with the prevalence of obesity-associated primary hypertension being 65–75% [15,16]. Furthermore, medical treatment of hypertension is more complex in patients with obesity, and often a combination of more than one pharmacological agent is needed to sufficiently control hypertension in this patient group [17].

Metabolic bariatric surgery (MBS) is the most effective treatment for severe obesity, leading to sustainable weight loss and often resulting in remission or alleviation of many obesity-related comorbidities [18,19]. MBS is associated with a reduced risk of cardiometabolic diseases and mortality, compared with routine care [20]. MBS reduces blood pressure [21] allowing many patients to discontinue antihypertensive medications after the surgery [22]. However, the role of MBS in preventing new-onset hypertension is poorly understood. The Swedish Obese Subjects (SOS) trial was the first to report that MBS significantly lowers the risk of new-onset hypertension, but the study utilized two surgical techniques that are no longer in current practice, and gastric bypass procedures represented only a minority of the operations [23]. Other studies assessing the preventive effect of MBS on new-onset hypertension are observational and mostly based on clinical cohorts [7]. The few follow-up studies on population-based cohorts have reported a reduced risk of new-onset hypertension after MBS compared to controls, with risk ratios ranging between 0.07 and 0.47 [20,22,24–26]. However, an important limitation of these studies is the lack of control for potential confounders such as socioeconomic status or comorbid conditions. Additionally, the effect of baseline obesity class on the comparative risk reduction of hypertension after MBS remains unknown.

In this study, we compared the risk of new-onset hypertension in patients who underwent MBS to propensity score-matched controls, taking into account a wide range of potential confounders such as sociodemographic factors, clinical characteristics, and residential environmental features. The aim was also to assess and compare the risk of new-onset hypertension in sociodemographic subgroups based on age, sex, socioeconomic status, and BMI classes [BMI 30–34.9 kg/m^2^ (Class I), 35–35.9 kg/m^2^ (Class II), and ≥40 kg/m^2^ (Class III]).

## METHODS

### Study population

The patients who underwent MBS and their matched controls were drawn from the Finnish Public Sector (FPS) study, a large, dynamic cohort of public sector employees with repeated questionnaire follow-ups every 2–4 years and linkage to electronic health records. The FPS was established in 1997–1998 and it comprises employees with a job contract for a minimum of six months in the municipal services of the six largest cities, five smaller towns, and 21 public hospitals in Finland [27]. A total of 477 509 individuals participating in the FPS had data on sociodemographic factors and were successfully linked to national health records until December 31, 2016.

After the entry to the cohort, all individuals in the FPS cohort who underwent laparoscopic MBS procedure, either a Roux-en-Y gastric bypass (LRYGB) or sleeve gastrectomy (LSG) between 2008 and 2016, were included in the present study. Information was collected from the national registry of hospital discharge records maintained by the National Institute for Health and Welfare. To define an eligible comparison group for propensity score matching, we identified all cohort participants who had been hospitalized with a diagnosis of obesity (ICD-10 code E66) for reasons other than MBS or who had a BMI ≥35 kg/m^2^ based on self-reported body height and weight in any of the three surveys conducted between 2008 and 2014. In the sensitivity analysis, a BMI range of 30–34.9 kg/m^2^ was also applied. Individuals with hypertension were excluded from the study. The propensity score was calculated for undergoing surgery based on a wide range of risk factors among this sample of hypertension-free participants with obesity. Each bariatric surgery case was then matched 1 : 2 to the nonsurgery comparison group with the same propensity score, leaving only nine bariatric patients unmatched.

The ethics committee of the Hospital District of Helsinki and Uusimaa approved the study (registration number HUS/1210/2016).

### Ascertainment of hypertension

Participants were linked to nationwide health and population registries using unique personal identification numbers in Finland. Hypertension was identified with eligibility for special reimbursement in the Drug Prescription Register and with ICD-10 code I10 in the hospital discharge registry, as in our previous studies [28]. Finnish National Health Insurance, coordinated by the Social Insurance Institution, covers all permanent residents of Finland and provides special reimbursements for additional costs associated with many chronic diseases, including hypertension. Participants with hypertension who required continuous medication were identified from the Social Insurance Institution Drug Reimbursement Register. This register contains data on all patients who, based on medical certificates, have been granted reimbursement for medications in Finland, including antihypertensive drugs, with the date on which permission was granted. To be eligible for this reimbursement due to hypertension, the patient needed to have a severe (at least stage 2) or complicated form of hypertension that is likely to be chronic (repeated blood pressure measurements of >105 mmHg diastolic (or >95 mmHg diastolic with signs of complications or cardiovascular co-morbidities) or >200 mmHg systolic). Follow-up started at the date of MBS for cases and the date of the recording of obesity (from hospital records or survey responses) for controls and lasted until December 31, 2016.

### Covariates

Baseline covariates were obtained via record linkage by the unique identification number of participants to national registries, as described in detail elsewhere [29]. Age, sex, and occupational titles were derived from employers’ records, and educational attainment was obtained from Statistics Finland. Socioeconomic status was defined by occupational position, educational attainment, and level of neighborhood disadvantage with detailed definitions published previously [27]. Occupational position was based on occupational titles categorized as higher-grade nonmanual, lower-grade nonmanual, and manual jobs. Educational attainment was categorized into tertiary (college or university), secondary (vocational), or basic. The geocoded residential addresses of the participants were obtained from the Population Register Center and positioned in the Statistics Finland 250 m × 250 m Grid Database [30]. The neighborhood disadvantage score for each grid comprised the average annual income of households, the mean number of years of education of those over 18 years of age, and the proportion of unemployed adults belonging to the labor force. The disadvantage score was classified into three categories based on national means as follows: affluent (deprivation score in relation to the national mean disadvantage score < −0.5 SD), intermediate (−0.5 to 0.5 SD), and disadvantaged (>0.5 SD, highest disadvantage). Low socioeconomic status was indicated by any of the following: basic education, manual occupation, or residence in a disadvantaged neighborhood. High socioeconomic status was indicated by a combination of tertiary or higher education, higher-grade nonmanual occupation, and residence in an affluent neighborhood. Intermediate socioeconomic status was defined as all other combinations. Based on records of granted work disability pensions and statutory pensions obtained from the Finnish Centre of Pensions, the participants were classified as not retired, retired due to work disability, and retired based on age. Information on chronic medical conditions was obtained from the national health registries. Healthcare provider-related differences were indicated by place of residence (city, town, or rural) and hospital district (five districts).

### Statistical analysis

The propensity score method was used to approximate the comparability of the cases and controls. The propensity score (PS) is the conditional probability of being assigned ‘treatment,’ here MBS, given the observed covariates (for details, see [29]). We constructed for all bariatric surgery patients and their nonsurgery comparison group using a logistic regression model with bariatric surgery as the outcome, and status regarding sex (female and male), age-group (18–35, 35–44, 45–54, 55–64 and >65 years), socioeconomic status (low, intermediate, or high), retirement, diagnosed medical conditions, place of residence, and hospital district defined prior to the index date as covariates. The model also included interactions of covariates with sex, age group, and socioeconomic status. Each bariatric surgery case was matched 1 : 2 with nonsurgery controls with the same propensity score. The degree of imbalance between the patients with MBS and their control persons was assessed using the standardized mean difference (SMD) [31]. For each covariate, the standardized mean difference (SMD) was calculated using the following formula:


$$SMD=100\times \left(Mb-Mc\right)/\sqrt{\left(\left(Vb+Vc\right)/2\right)},$$

where Mb and Mc represent the sample means for the bariatric surgery and control groups, respectively, and Vb and Vc are the corresponding sample variances. Absolute SMD values below 10% indicate a good balance between the groups.

To examine the risk of new-onset hypertension among bariatric surgery cases and their matched controls, we started the follow-up at the index date: hospitalization due to bariatric surgery, hospitalization with an ICD-10 code of obesity but no bariatric surgery, or the date of survey response with a BMI ≥35 kg/m^2^ for those with no hospitalization with an ICD-10 code of obesity. The follow-up continued until disease onset, death, or the end of follow-up (December 31, 2016), whichever came first.

To depict the association of bariatric surgery with new-onset hypertension during the follow-up period, we calculated the cumulative hazard curves for bariatric surgery cases and their matched controls. We used Poisson regression models to estimate the rate of new-onset hypertension per 1000 person years and the 95% confidence intervals (95% CI) in the bariatric surgery group and the control groups, and the corresponding rate ratio and rate difference (95% CI). To examine whether the associations varied between demographic subgroups, we used contrasts in the Poisson regression models. These included the demographic factor, treatment group, and their interaction term to estimate the rates, rate ratios, and rate differences for men and women, for those aged less than 50 years and 50 years or more, and for those with low socioeconomic status.

In sensitivity analyses, we first calculated the cumulative hazard curves and estimated the rates, rate ratios, and rate differences, as in the main analyses comparing the risk of MBS patients to four alternative propensity score-matched control groups (clinical controls only and nonclinical survey controls with BMI ≥40, 35–39.9, or 30–34.9 kg/m^2^), matched 1 : 1. Second, we replicated the main analyses using a minimum 1-year lag between the index date and the date of the occurrence of hypertension to consider the possibility that nondiagnosed prevalent hypertension would be more likely among the nonsurgery controls than the bariatric patients. Third, we examined the association between MBS and hypertension onset according to the type of surgery (LRYGB or LSG). All analyses were performed using SAS version 9.4.

## RESULTS

**FIGURE 1:**
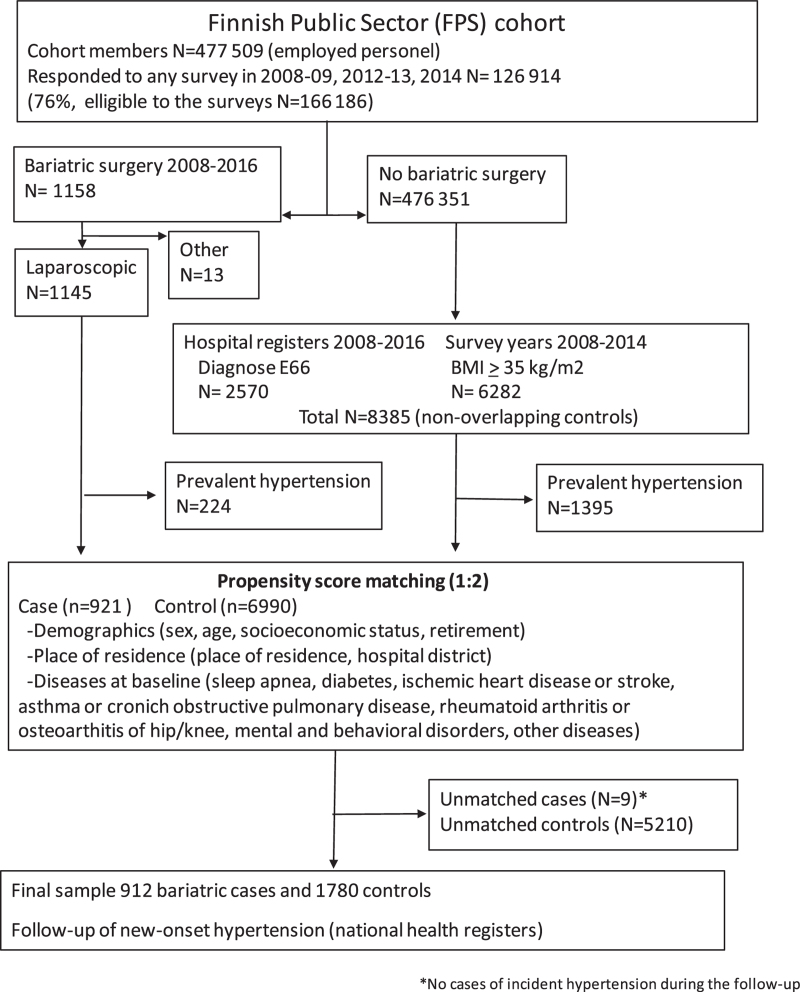
Flow chart of the study population.

A total of 1145 patients underwent laparoscopic Roux-en-Y gastric bypass (LRYGB) or sleeve gastrectomy (LSG). There were 8385 potential controls with no history of bariatric surgery who had been hospitalized with a diagnosis of obesity or who had a self-reported BMI ≥35 kg/m^2^. For sensitivity analyses, BMI Class I (30–34.9 kg/m^2^) was also applied. The selection of the analytical samples is shown in Fig. 1. After excluding individuals with prevalent hypertension at baseline (224 bariatric surgery patients and 1395 controls, prevalence 19.6% and 16.6%, respectively), each bariatric surgery case was matched to two nonsurgery comparison patients with the same propensity score, leaving only nine unmatched MBS patients. Of the nine unmatched patients, none had new-onset hypertension during follow-up. The final sample consisted of 912 patients with MBS cases and 1780 matched controls. The distributions of the baseline characteristics of the MBS cases and their controls are shown in Table 1. The mean age of the 2692 participants was 44.5 years, 2469 (91%) were women, and 1399 (52%) had low socioeconomic status. The absolute SMD values for all baseline characteristics were small (<10%), supporting the assumption of balance between the groups (Table 1).

**TABLE 1:** Baseline characteristics of propensity-score-matched cohort of patients with bariatric surgery and their control persons

Controls have been hospitalized with the diagnosis of obesity (ICD-10 E66) or have BMI ≥35 m^2^ but have no history of bariatric surgery			
Characteristic	Controls	Bariatric	SMD (%)
All, *N* (%)	1780 (100)	912 (100)	
Sex, *N* (%)			2.1
Men	144 (8.1)	79 (8.7)	
Women	1636 (91.9)	833 (91.1)	
Mean age (SD), years	44.5 (9.9)	44.6 (9.4)	−1.4
Socioeconomic status, *N* (%)			
Low	923 (51.9)	476 (52.2)	−0.7
Intermediate	685 (38.5)	348 (38.2)	0.7
Higher grade nonmanual	172 (9.7)	88 (9.7)	0.0
Retirement, *N* (%)			
Not retired	1496 (84.0)	760 (83.3)	1.9
Disability retirement	279 (15.7)	148 (16.2)	−1.5
Statutory retirement	5 (0.3)	4 (0.4)	−2.6
Diseases, *N* (%)			
Sleep apnea	236 (13.3)	138 (15.1)	−5.4
Diabetes	186 (10.5)	110 (12.1)	−5.1
Cardiovascular diseases^a^	43 (2.4)	25 (2.7)	−2.1
Asthma or COPD	266 (14.9)	132 (14.5)	1.3
Musculoskeletal disorders^b^	178 (10.0)	99 (10.9)	−2.8
Mental or behavioral disorders	167 (9.4)	80 (8.8)	2.1
Other diseases^c^	61 (3.4)	38 (4.2)	−3.9
Place of residence			
City	898 (50.5)	453 (49.7)	1.6
Town	494 (27.8)	259 (28.4)	−1.4
Rural	388 (21.8)	200 (21.9)	−0.3
Hospital district			
Helsinki and Uusimaa	695 (39.0)	331 (36.3)	5.7
South-West Finland	273 (15.3)	160 (17.5)	−6.0
Pirkanmaa	280 (15.7)	154 (16.9)	−3.1
Northern Ostrobothnia	202 (11.4)	103 (11.3)	0.2
Other	330 (18.5)	164 (18.0)	1.4

SMD, standardised mean difference.

a IHD, heart failure, stroke.

b Rheumatoid arthritis and related disorders, osteoarthritis of hip, osteoarthritis of knee.

c Parkinson disease, multiple sclerosis, epilepsy, alcoholic liver disease, pancretitis, renal failure.

**FIGURE 2:**
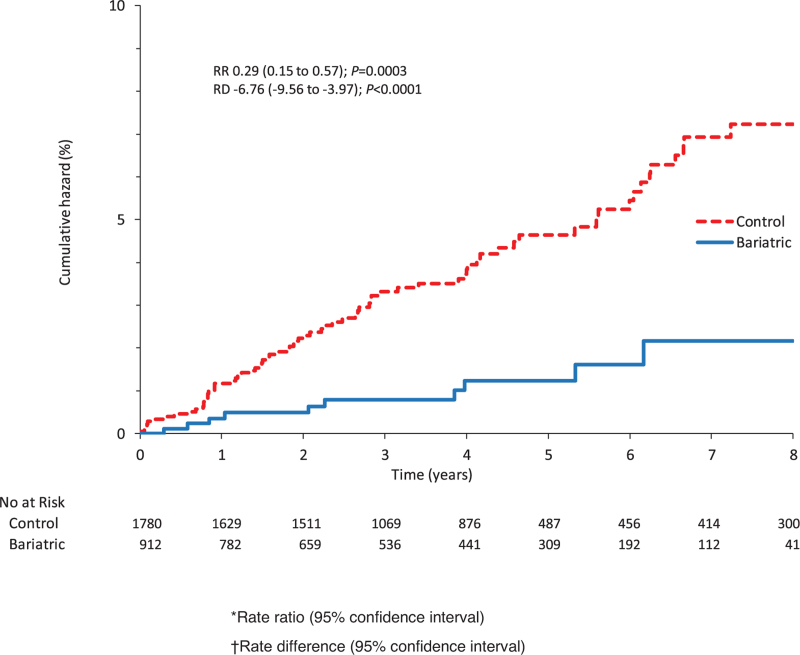
Eight-year cumulative hazard (%) of hypertension onset in bariatric cases and matched controls.

**FIGURE 3:**
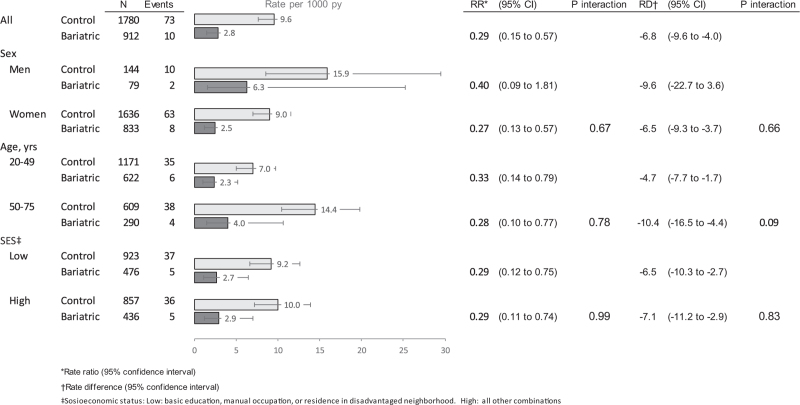
Bariatric surgery and risk of new-onset hypertension compared to matched controls.

During the mean follow-up of 4.2 years (range 0–9 years), new-onset hypertension cumulated linearly in both groups (Fig. 2). At the end of the follow-up, there were 83 new-onset hypertension events: 10 cases in the bariatric surgery group and 73 cases in the control group (Fig. 3). The rate of hypertension occurrence per 1000 person years was 9.6 (95% CI 7.6–12.0) in the control group and 2.8 (1.5–5.2) in the MBS group. The rate ratio in the bariatric cases compared to the controls was 0.29 (0.15–0.57) and the corresponding rate difference −6.8 (−9.7 to −3.8) per 1000 person-years.

No significant difference in the relative or absolute risk for hypertension after MBS was observed according to patient sex, age, or socioeconomic status (Fig. 3).

**FIGURE 4:**
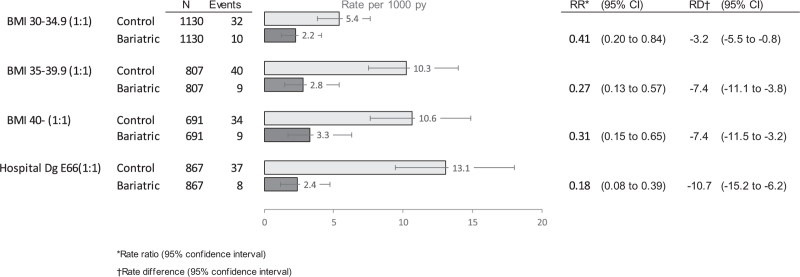
Bariatric surgery and risk of new-onset hypertension compared to alternative matched control groups.

Results from the sensitivity analyses with alternative matched control groups are shown in Fig. 4, and the cumulative hazard curves are shown in eFigure 1. Among the controls, the lowest obesity-related hypertension risk was observed in survey controls with BMI between 30 and 34.9 kg/m^2^ (5.4/1000 person-years) and the highest in hospital controls (13.1/1000 person-years). The relative risk of the MBS cases compared to these control groups varied in between 0.18 (hospital controls) and 0.41 (survey controls with BMI in between 30 and 34.9 kg/m^2^). The sensitivity analysis with a 1-year lag before the start of the follow-up of new-onset hypertension also replicated the findings of the main analyses (RR 0.28, 95% CI 0.13–0.62). The sensitivity analysis examining the association between MBS and hypertension by the type of surgery found no significant differences in the associations with new-onset hypertension between LSG (*N* = 233) and RYGBP (*N* = 679). The corresponding rate ratios were 0.34 (0.11–1.09) and 0.28 (0.13–0. 60) years, respectively.

## DISCUSSION

In this follow-up study with propensity score-matched controls, we reported a significant reduction in the risk of new-onset hypertension following metabolic bariatric surgery. Risk reduction was also evident when comparing bariatric surgery cases to controls with Class I obesity (BMI 30–34.9 kg/m^2^), highlighting the efficacy of MBS in preventing new-onset hypertension. There were no statistically significant differences in the risk reduction associated with patient sex, age, or socioeconomic status.

The incidence rate of hypertension was 2.8 per 1000 person-years in the MBS group and 9.6 per 1000 person-years in the control group. This implies that approximately 150 patients with severe obesity should be treated by MBS to avoid one new case of hypertension in a year. However, the clinical significance is much higher as MBS reduces the risk of a wide range of obesity-related conditions in addition to hypertension.

Metabolic bariatric surgery has been shown to result in sustainable weight loss and remission or alleviation of many obesity-related diseases, such as hypertension and T2DM [18,19]. However, the extent to which MBS prevents new-onset hypertension is not well known. Our results are in line with the previous follow-up studies on population-based cohorts that have examined the effect of MBS on the risk of new-onset hypertension, which was found to be 60–90% lower in patients compared to controls (pooled odds ratio 0.36, 95% CI 0.32–0.40) [7,20,22,24–26]. These studies were extensive nonrandomized comparative studies reliant on registry data with 956–8385 MBS patients with a maximum follow-up time from 6 to 15 years. However, there were major limitations to these studies. In only two studies, the definition of hypertension was based on a clinical diagnosis derived from patient care registries, as was the case in our study [20,25]. In other studies, a surrogate marker of hypertension, the Anatomical Therapeutic Chemical (ATC) codes used for antihypertensive drugs, was used, with the onset of hypertension defined as the first time a participant had a drug dispensed in electronic pharmacy claim records [24,26], or the first year a participant had had three such purchases [22]. This creates a potential source of error as the indication for the prescription of antihypertensive drugs is unknown, and their usage is not restricted to hypertension. Similar to our study, four prior studies used propensity score matching, but only used patient age and sex [20,22,24,25] and additionally baseline diabetes [20,22,24] or data from general practitioners [25] to match controls with obesity with the MBS patients. Socioeconomic status was not controlled for in these studies with two exceptions [20,22] and baseline co-morbidities outside T2DM were not taken into account. As our study setting allowed for the control of a wide range of potential confounders, we were able to confirm and expand on the results of prior studies.

In contrast to our previous study, in which we found that the risk of new-onset T2DM varied across socioeconomic statuses following MBS (see ref. [29]); no such differences were observed in this study. A potential explanation is that hypertension is more multifactorial, and the risk of hypertension is associated with for example. genetics, age, and behavioral factors such as low physical activity, obesity, high-salt diet, excess alcohol intake and smoking [2]. MBS exerts its effects on blood pressure through numerous mechanisms, such as weight loss, reduced sympathetic activity, improved insulin sensitivity, lower renin–angiotensin–aldosterone system activation and decreased chronic low-grade inflammation [16]. In addition, hypertension is an integral component of the cardiovascular risk profile. In particular, coexisting with T2DM can synergistically increase the risk of cardiovascular complications. The management or prevention of both T2DM and hypertonia is crucial for reducing the overall cardiovascular risk, and MBS addresses both conditions.

Hypertension can be effectively managed pharmacologically, but the efficacy of the treatment is often limited by poor patient adherence to long-term antihypertensive medication, and the benefits are lost upon discontinuation. For example, prevalence of good adherence to antihypertensive medication was only 59% (95% CI, 42–77) [32]. At the same time, new obesity management medications (OMMs) are emerging as alternatives to MBS as a means to reduce both weight and obesity-related cardiovascular risk factors. Recent findings in the SELECT study suggest that semaglutide significantly reduces the 5-year risk of cardiovascular events in obese patients. However, discontinuation of the medication was not uncommon and resulted in recurrent weight gain and a return of previously improved cardiovascular risk factors to baseline levels [33,34]. The need for life-long medication to sustain the positive effects of OMMs is associated with significant costs, problems with medication adherence and availability, and potential adverse effects [34,35]. In contrast, the results of MBS are often long-lasting, and the overall costs are likely lower than those for continuous OMM treatment. However, OMMs can offer an alternative treatment for severe obesity in those who are not suitable candidates for MBS [36,37]. or they can be used for synergy in conjunction with MBS [38,39].

This study had several limitations. First, the selection of controls was based on either the diagnosis of obesity in the hospital discharge registry or self-reported height and weight, with self-reported measurements as a potential source of bias. However, the cumulative hazard curves of new-onset hypertension in controls with class I, II, and III obesity followed a dose-response pattern. This and our earlier studies suggest that self-reported data did not cause a significant bias [27]. Second, we identified hypertension, a condition often asymptomatic, with either eligibility for special reimbursement in the Drug Prescription Register or ICD-10 code I10 in the hospital discharge registry. Considering that individuals who undergo MBS are usually carefully evaluated by a multidisciplinary team, it is unlikely that hypertension remained undiagnosed in the surgery group. In contrast, it is possible that, among the survey controls, there were undiagnosed cases of baseline hypertension. However, sensitivity analyses showed that the cumulative hazard curves for hypertension were similar in shape in clinical controls compared with survey controls, where obesity was measured using self-reported weight and height. Moreover, the results remained the same using a minimum 1-year lag between the index date and the date of the occurrence of hypertension, mitigating the possibility that nondiagnosed prevalent hypertension would have biased this study. Third, information about behavior-related risk factors for hypertension, such as smoking or alcohol intake, was not available. These factors might affect the likelihood of seek MBS or being suitable for MBS. However, we were able to use information on hospitalizations due to mental and behavioral disorders, a correlate of the use of addictive substances, diminishing the possibility that substance abuse would be a major source of bias in our study [27]. Fourth, nine patients with MBS were excluded because there were no potential controls with the same propensity score. As none of the patients developed new-onset hypertension, this exclusion had little effect on our results. Fifth, in our female dominated cohort, more than 90 percentages of our participants were women, which is consistent with MBS showing a predominance of female patients worldwide [40]. Although there were no statistically significant differences in the risk reduction associated with patient sex, we cannot rule out the possibility that the risk reduction after MBS would vary by sex given the small number of men in our study. Finally, our study was based on an ethnically homogenous occupational cohort in a single welfare country with universal healthcare, and further studies are needed to assess the effect of MBS outside of employed population segments and in various ethnic groups.

Our study had significant strengths. Patient characteristics, such as socioeconomic status and comorbidities, the timing of MBS, and the detection of new-onset hypertension, were derived from reliable national registries with no loss of follow-up. The treatment and comparison groups were well balanced by propensity score matching with a wide range of potential confounding factors, including demographics, socioeconomic factors, clinical characteristics, and living environment features. Our study was performed in a Scandinavian welfare country with universal healthcare, diminishing the selection bias related to socioeconomic status.

In conclusion, our study confirmed that metabolic bariatric surgery markedly reduced the risk of new-onset arterial hypertension and showed that there were no statistically significant differences in this reduction across all age, sex, and socioeconomic subgroups. Our findings underline the need for increased access to MBS to effectively treat severe obesity and prevent the development of hypertension, which is one of the leading causes of cardiovascular death.

## ACKNOWLEDGEMENTS

Funding: Viiko Vahtera was supported by ERVA research funding from the Hospital District of Southwest Finland (VTR 13136), Finnish Society of Gastrointestinal Surgeons, Finnish Association of Bariatric and Metabolic Surgery (LIME), Doctoral Program in Clinical Research of the University of Turku, Turku, Finland. Mika Kivimäki was supported by the Wellcome Trust (221854/Z/20/Z), UK Medical Research Council (MRC S011676/1, R024227/1), US National Institute on Aging (R01AG056477), Academy of Finland (350426), and Finnish Foundation for Cardiovascular Research (a86898). Jenni Ervasti was supported by the Academy of Finland, Strategic Research Council (358458), and Finnish Work Environment Fund (220245). Sari Stenholm was supported by the Academy of Finland (332030). Jussi Vahtera was supported by Academy of Finland (329240). Paulina Salminen was supported by the Sigrid Jusélius lius Foundation.

Disclosure: Dr Paulina Salminen reports on lecture fees from Novo Nordisk. All other authors declare no competing interests.

### Conflicts of interest

There are no conflicts of interest.

## Supplementary Material

Supplemental Digital Content

## Data Availability

Anonymized questionnaire data from the Finnish Public Sector Study can be shared by request with the investigators after approval of the Finnish Institute of Occupational Health (contact E-Mail: jenni.ervasti@ttl.fi). Linked electronic health records require separate permission from the National Institute of Health and Welfare and Statistics, Finland.
