# Supplementary material for: Incidence of new onset arterial hypertension after metabolic bariatric surgery: an 8-year prospective follow-up with matched controls
Source: J Hypertens. 2025 Mar 10;43(5):871–9. doi: 10.1097/HJH.0000000000003993 (PMC11970605; doi:10.1097/HJH.0000000000003993)

eFigure 1. Eight-year cumulative hazard (%) of hypertension onset in alternative matched control groups and bariatric surgery patients


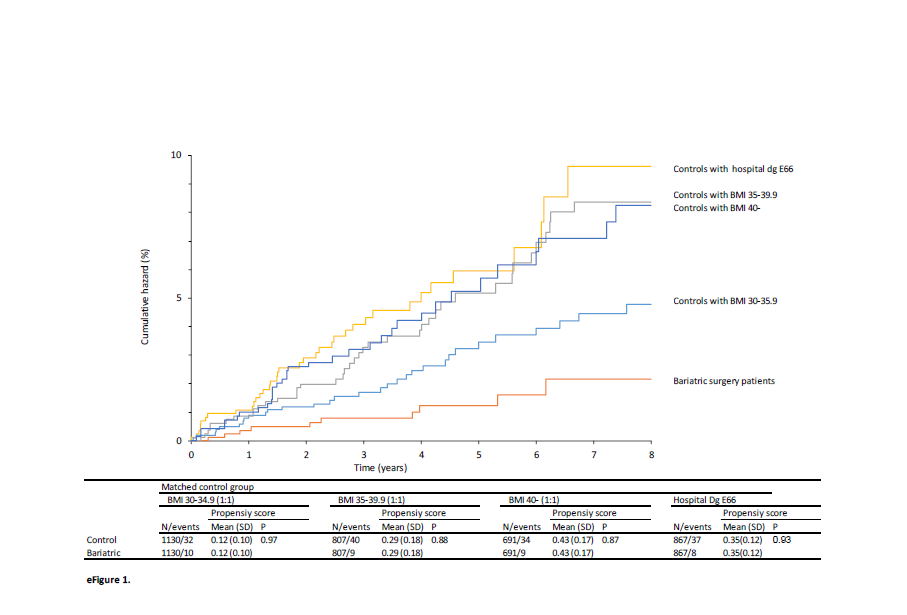

Supplement: Supplemental Digital Content [file jhype-43-871-s001.doc]
